# Successful Treatment of Type 3b Endoleak after AFX Using TREO

**DOI:** 10.3400/avd.cr.25-00016

**Published:** 2025-06-05

**Authors:** Tobuhiro Nita, Hironori Baba, Yuji Hironaka, Gen Shinohara, Yoshie Ochiai, Shigehiko Tokunaga

**Affiliations:** Department of Cardiovascular Surgery, JCHO Kyushu Hospital, Kitakyushu, Fukuoka, Japan

**Keywords:** EVAR relining, type 3 endoleak, TREO

## Abstract

Late postoperative type 3 endoleaks associated with the AFX (Endologix, Irvine, CA, USA) stent-graft are commonly reported. The AFX’s endoskeletal design raises concerns about wire entrapment between its frame and fabric, as well as a risk of type 1a endoleak. An 84-year-old man with prior EVAR using the AFX presented with a type 3b endoleak 4 years postoperatively. He underwent relining with the TREO stent-graft (Terumo Aortic, Sunrise, FL, USA), which enabled easy contralateral gate cannulation and secured a long proximal landing zone. The TREO appears to be a viable option for relining the AFX in type 3b endoleak cases.

## Introduction

AFX (Endologix, Irvine, CA, USA) has been reported to exhibit a higher frequency of type 3 endoleaks in the long-term follow-up phase.^1,2)^ Type 3 endoleaks have been associated with aneurysm enlargement and increased mortality, necessitating aggressive intervention. There are, however, concerns about wire and device entrapment between the wire frame and the graft fabric due to the AFX’s endoskeletal design. The TREO stent-graft (Terumo Aortic, Sunrise, FL, USA) features a long main body, which facilitates securing the device and deploying the opposite leg gate closer to the terminal aorta. We hypothesized that this feature could simplify contralateral gate cannulation and reduce the risk of wire entrapment. The patient provided written informed consent for the report of his case details and imaging studies.

## Case Report

**Figure 1:**
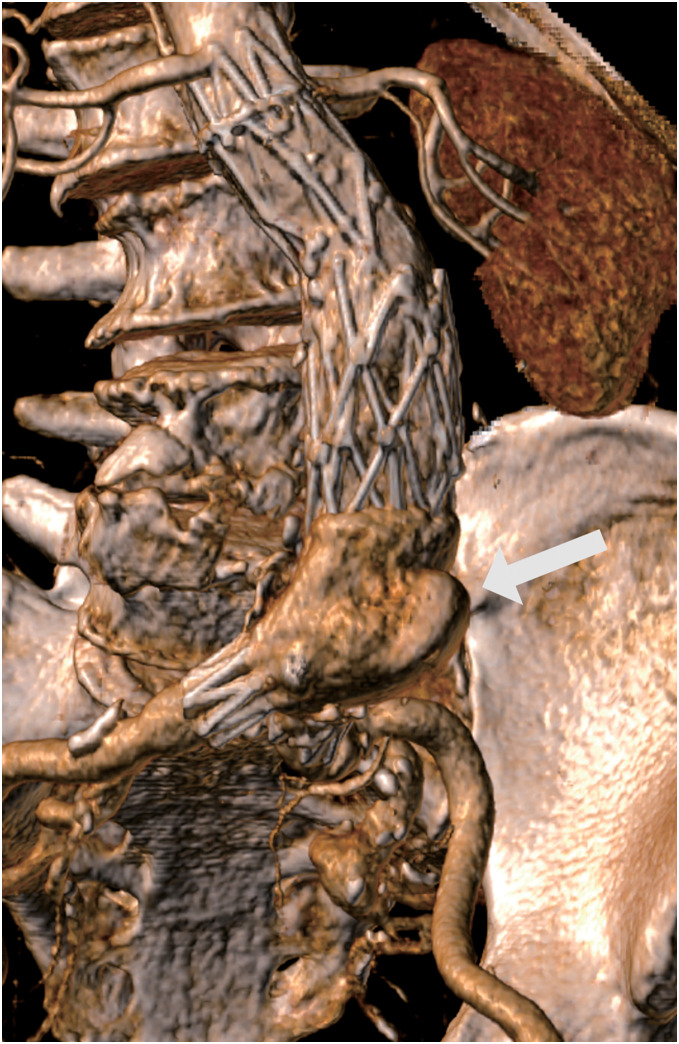
Fig. 1 Preoperative CT image. Preoperative contrast-enhanced computed tomography (CT) images of the abdominal aorta 3 years after AFX implantation. A type 3 endoleak was suspected (arrow).

**Figure 2:**
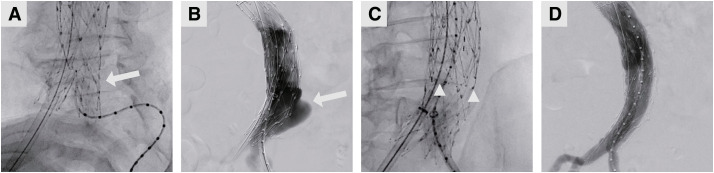
Fig. 2 Intraoperative angiogram of a patient during endovascular aneurysm repair (EVAR). (**A**) Cannulation of the AFX endograft with a pigtail catheter, with the tip curved (arrow). (**B**) Contrast enhancement in the AFX endograft demonstrated a type 3b endoleak (arrow). (**C**) Cannulation of the contralateral leg of the TREO graft, with its gate positioned above the DrySeal sheath (the gate was between the triangles). (**D**) Final contrast injection showed no type 3 endoleaks.

An 84-year-old man with an abdominal aortic aneurysm, diagnosed 4 years ago, was scheduled for surgery due to a 5-mm enlargement of the aneurysm, from 44 mm to 49 mm, over a 6-month period. In the same year, he underwent coronary artery bypass surgery (LITA-LAD, Ao-SVG-3rd-PL), followed by EVAR (AFX), resulting in regression of the postoperative aneurysm sac. A non-contrast CT performed 4 years later showed aneurysm dilation from 40 mm to 45 mm, while the overlap length between the proximal extension and the main body remained unchanged at 54 mm. A contrast-enhanced CT subsequently revealed a type 3b endoleak at the site of limb bifurcation (**Fig. 1**). He underwent relining of the EVAR to prevent aneurysmal rupture. Following bilateral inguinal incisions, the common femoral artery was secured, and an 8Fr sheath was inserted. The guidewire was stored in the pigtail catheter in the AFX open-leg position and inserted into the device with the tip curved (**Fig. 2A**). After the guidewire and catheter were routed to the descending aorta, the guidewire was replaced with a stiff wire, and balloon dilation was performed to confirm that the wire was not entrapped between the skeleton and the fabric. Contrast imaging of the graft confirmed the presence of a type 3b endoleak (**Fig. 2B**). The proximal end of the TREO device was deployed to align with the proximal end of the AFX, and the gate on the opposite side was released. The contralateral gate was positioned directly above the sheath, facilitating easy cannulation (**Fig. 2C**), and balloon dilation ensured there was no device entrapment between the wire frame and the fabric of the AFX. Final contrast imaging demonstrated the disappearance of the endoleak (**Fig. 2D**). The patient had an uneventful postoperative course, and contrast-enhanced CT on postoperative day 5 confirmed the disappearance of the endoleak.

## Discussion

According to Forsyth et al., the median time to occurrence of type 3 endoleaks after AFX implantation was 4.7 years, even in the long-term phase, with no significant difference in the frequency of type 3a and type 3b endoleaks.^1)^ Lemmon et al. stated that most type 3b endoleaks from Endologix endografts result from fabric tears that propagate from the bifurcation of the main body.^3)^ Although these reports pertain to the previous generation of AFX, type 3b endoleak resulting from structural features is equally concerning. The design of the AFX graft puts it at risk for repetitive microtrauma or shear injury due to its aortic bifurcation, which is often heavily calcified. This configuration, combined with the advertised thinner fabric design of the Strata material, may predispose the graft to type 3b endoleaks.^1)^

When a type 3 endoleak occurs, reintervention should be considered because the treated aortic aneurysm remains pressurized, and the therapeutic effect of EVAR is not achieved.^4)^ Relining has been reported as an effective method for reintervention; however, surgical explantation may be necessary if relining is incomplete or if the endoleak persists after relining.^1)^ In this case, relining was performed according to the preoperative plan, and no residual endoleaks were observed.

Because the AFX has an endoskeletal design, wires can become entrapped between the skeleton and the fabric. Some reports have used balloon dilation to confirm the absence of wire entrapment, but this technique is tedious and time-consuming. Surgical complexity is particularly high in cases where cannulation of the contralateral gate is challenging. There are few reports of relining for type 3b endoleaks associated with AFX; however, successful cases have been reported using devices from Gore, Medtronic, and Endologix.^1)^ There is no clear evidence about the optimal EVAR device for relining AFX.

TREO is a new generation of endoprosthesis, characterized by a long main body.^5,6)^ This design has two advantages. First, the TREO has a long covered length from the proximal edge of the fabric to the contralateral limb stump, measuring 100 mm (only the 100 mm graft is used in Japan), which is longer than other EVAR devices. If the distance from the terminal aorta to the contralateral gate is short, and the direction of the gate opening aligns with the sheath, cannulation can be performed easily. Simplifying contralateral gate cannulation is an effective method for preventing entrapment between the AFX endoskeleton and the fabric. Second, the TREO has a longer body (70 mm) than other EVAR devices, allowing for a longer proximal landing zone. Selecting a long endograft is advisable, as placing an exoskeletal device, such as the TREO or similar devices, inside an endoskeletal-designed AFX carries a high risk of type 1a endoleak.

## Conclusions

This case demonstrates that the TREO stent-graft can be an effective option for relining AFX in the treatment of type 3b endoleak. Its structural features may help reduce technical challenges and improve outcomes, though further studies are needed to confirm its role in reintervention strategies.

## Declarations

### Conflicts of interest

All authors have declared no conflict of interest.

### Author contributions

Study conception: TN, HB

Manuscript preparation: TN, HB

Critical review and revision: all authors

Final approval of the article: all authors

Accountability for all aspects of the work: all authors.
